# A randomised trial of granulocyte-macrophage colony-stimulating factor for neonatal sepsis: childhood outcomes at 5 years

**DOI:** 10.1136/archdischild-2014-307410

**Published:** 2015-04-28

**Authors:** Neil Marlow, Timothy Morris, Peter Brocklehurst, Robert Carr, Frances Cowan, Nishma Patel, Stavros Petrou, Margaret Redshaw, Neena Modi, Caroline J Doré

**Affiliations:** 1 UCL Institute for Women's Health, University College London, London, UK; 2 MRC Clinical Trials Unit, University College London, London, UK; 3 Department of Haematology, Guy's and St Thomas’ Hospital, King's College London, London, UK; 4 Section of Neonatal Medicine, Department of Medicine, Chelsea & Westminster campus, Imperial College London, London, UK; 5 Department of Applied Health Research, University College London, London, UK; 6 Warwick Medical School, University of Warwick, Coventry, UK; 7 National Perinatal Epidemiology Unit, University of Oxford, Oxford, UK

**Keywords:** Child Psychology, Haematology, Neonatology, Neurodevelopment

## Abstract

**Objective:**

We performed a randomised trial in very preterm, small for gestational age (SGA) babies to determine if prophylaxis with granulocyte macrophage colony stimulating factor (GM-CSF) improves outcomes (the PROGRAMS trial). GM-CSF was associated with improved neonatal neutrophil counts, but no change in other neonatal or 2-year outcomes. As subtle benefits in outcome may not be ascertainable until school age we performed an outcome study at 5 years.

**Patients and methods:**

280 babies born at 31 weeks of gestation or less and SGA were entered into the trial. Outcomes were assessed at 5 years to determine neurodevelopmental and general health status and educational attainment.

**Results:**

We found no significant differences in cognitive, general health or educational outcomes between 83 of 106 (78%) surviving children in the GM-CSF arm compared with 81 of 110 (74%) in the control arm. Mean mental processing composite (equivalent to IQ) at 5 years were 94 (SD 16) compared with 95 (SD 15), respectively (difference in means −1 (95%CI −6 to 4), and similar proportions were in receipt of special educational needs support (41% vs 35%; risk ratio 1.2 (95% CI 0.8 to 1.9)). Performance on Kaufmann-ABC subscales and components of NEPSY were similar. The suggestion of worse respiratory outcomes in the GM-CSF group at 2 years was replicated at 5 years.

**Conclusions:**

The administration of GM-CSF to very preterm SGA babies is not associated with improved or more adverse neurodevelopmental, general health or educational outcomes at 5 years.

**Trial registration number:**

ISRCTN42553489.

**What is already known on this topic?**

- Neonatal neutropenia and sepsis are common following fetal growth restriction.
- Administration of granulocyte macrophage colony stimulating factor (GM-CSF) after birth raises neutrophil counts but does not improve neonatal or 2-year developmental outcomes.

**What this study adds?**

- Granulocyte macrophage colony stimulating factor (GM-CSF) administered after birth does not alter 5-year neurocognitive outcomes.
- Preterm small-for-gestational-age (SGA) babies have a higher prevalence of special educational needs than anticipated from their gestational age at birth.

## Introduction

Perinatal infection and inflammation have been implicated in the causal pathway of neonatal preterm brain injury and deviations from normal neurodevelopmental patterns.^1^ ^2^ Many developmental problems, such as learning difficulty, poor general and executive cognitive processes and behavioural disorders, are not easily determined until early school age. Interventions targeted at reducing the inflammatory load during the neonatal period may therefore not demonstrate benefit until such functions are measureable.

Very preterm babies with fetal growth restriction, who are born small for gestational age (SGA), are at high risk of developing acquired sepsis after birth and this has been believed to relate to the frequent observation of neutropenia in such babies. Granulocyte macrophage colony stimulating factor (GM-CSF) has been shown to be effective at reducing neutropenia-related infections in patients with cancer after chemotherapy.^3^ In order to study the potential benefits of GM-CSF in preterm growth-restricted infants, we undertook PROGRAMS, a single blind, multicentre, randomised trial of GM-CSF in very preterm SGA babies, to determine whether treatment resulted in a reduced incidence of infection, mortality and morbidity in the neonatal period and over the longer term. Two hundred and eighty newborn SGA infants of 31 completed weeks gestational age or less were randomly allocated to GM-CSF or routine treatment within 72 h of birth. Although neutrophil counts were significantly higher in GM-CSF treated babies, there was no significant difference in sepsis-free survival at 14 days from trial entry between the two treatment arms,^4^ in keeping with the findings of our updated systematic review,^5^ and no benefit was demonstrated in terms of developmental outcomes at 2 years of age adjusted for prematurity.^6^ However, we observed a larger proportion of children with developmental impairment than was consistent with the degree of prematurity and speculated that intrauterine growth restriction placed these children at particular developmental risk.

As part of the original design of the trial, we hypothesised that there might be more subtle benefits over and above the relatively short-term developmental outcomes described above and therefore designed an outcome evaluation at 5 years of age to determine whether the administration of GM-CSF in the neonatal period was associated with differences in the prevalence of developmental problems.

## Methods

Full details of the PROGRAMS trial^4^ and 2-year follow up^6^ have been published. Briefly, participants were infants born at ≤31 completed weeks of gestation with birth weight <10th centile (UK 1990 Growth Reference). An infant was not eligible if there was an immediately life-threatening congenital abnormality, or a strong likelihood of early onset sepsis, indicated by maternal pyrexia exceeding 38°C on two occasions during labour. The study intervention, GM-CSF, was given in a dose of 10 µg/kg subcutaneously daily for five consecutive days. No placebo injections were administered to the standard treatment arm of the study. Two commercial preparations of recombinant human GM-CSF were used during the study, molgramostim (Leucomax, Novartis, UK), and sargramostim (Leukine, Berlex, California, USA), which have equivalent biological potency for stimulating granulocyte production and function, in vitro and in vivo. Consistent with this, neutrophil counts were significantly higher in the treatment arm. At 2 years of age 94% of surviving children were evaluated.

## Five-year outcome evaluation

Contact was maintained with the families enrolled in the study following the 2-year evaluation; parents were informed of the results of the trial and follow-up study by newsletter. As previously, the children were traced and the families contacted by the study coordinator. Parents were asked for permission to carry out the evaluation at 5 years and to provide details of their child's school. Head teachers were then approached and a request made for a member of the study team to visit the school and carry out the testing. Parents were invited to attend as they wished. The assessment was carried out as close to the child's 5th birthday as feasible and no adjustment was made for gestational age at birth. Where a child was in a special school with serious disability, a formal assessment by a paediatrician and estimate of learning attainment was made. Details of school attainment and need for extra educational support (‘special needs’) were provided by the child's class teacher, who also completed the teacher's report for the Strengths and Difficulties Questionnaire (SDQ, http://www.sdqinfo.com) to evaluate behaviour at school.

Psychologists were trained and validated in the study evaluations. Children were assessed in school and the assessors masked to group assignment.

The primary outcome measure was general intelligence as measured using the mental processing composite (MPC; equivalent to IQ) of the Kaufmann-ABC (Pearson UK).^7^ The MPC and subscales were originally normed to a mean of 100 and a SD of 15 in the general population. The subscales used were simultaneous processing, sequential processing, maths and riddles. Executive functions were evaluated using components from NEPSY (Pearson, UK)^8^ in the domains of sensorimotor, visuospatial, attention-executive, language and memory/learning. Scaled scores taken from normative data were compared between trial groups. Behaviour was evaluated using parent and teacher report forms from the SDQ, generating overall scores (assessed against population norms to generate the proportion with disorder) and subscale scores for emotional symptoms, conduct problems, hyperactivity-inattention, peer problems and (for teachers’ report) school adaptation. Height, weight and head circumference were measured by the assessor using standard techniques (Leicester Height Measure (Seca), Scales (Salter Model 918) and Lass-O tapes (Child Growth Foundation)), and referred to UK-WHO Child Standards.^9^

In addition to the SDQ, parents also completed questionnaires detailing household socioeconomic status, and the language and communication element of the 4-year evaluation from the Twins and Early Development Study,^10^ hospitalisations since 2 years and a profile of respiratory symptoms and treatment derived from the ISAACS questionnaire.^11^

## Statistical methods

The sample size for the initial study was based on the short-term primary outcome, survival without sepsis for 14 days from trial entry.^4^

A CONSORT diagram was constructed, showing the flow of participants through the study.^12^ Variables were summarised as number (per cent) or median (25th–75th centile) for categorical or continuous/ranked data, respectively (none of the continuous variables approached approximate normality). For analysis of outcomes, relative risks were used to quantify the effect of treatment on categorical variables. Median differences between treatment groups were calculated for continuous and ranked data; 95% CIs were calculated to quantify uncertainty about relative risks and median differences.^13^

All analyses were conducted on an intention-to-treat basis, that is, participants were never excluded from analyses on the basis of the treatment received. The participants lost to follow-up or withdrawn were not included in analyses.

## Study management

Written informed consent was obtained from parents. Trial oversight was provided by an independent steering committee and independent data monitoring and ethics committee. This study is registered as an International Standard Randomised Controlled Trial, number ISRCTN42553489.

## Results

**Figure 1:**
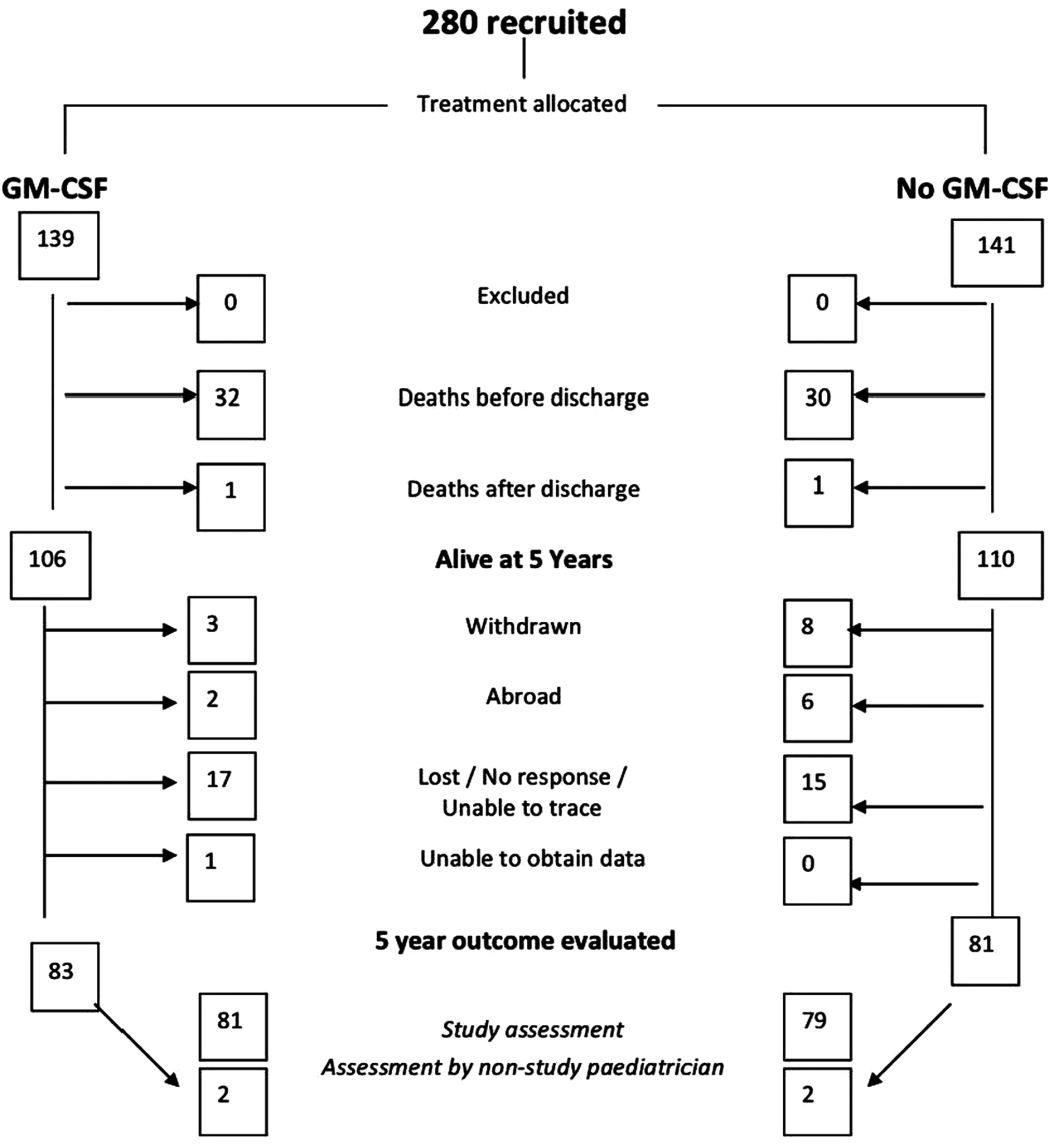
Consort diagram for the PROGRAMS study. GM-CSF, granulocyte macrophage colony stimulating factor.

Of 280 babies enrolled in the study, 279 completed the study intervention, 64 babies died (62 before and 2 after neonatal discharge), 11 were withdrawn by their parents and a further 41 families were lost to follow-up at 5 years. Details of the participant flow through the study are shown in figure 1. In the GM-CSF arm, 83 children were evaluated at a median of 65 months (range 50–78) and 81 in the control arm at a median of 65 months (52–78). There were no significant or systematic differences between those who were included and those who dropped out of the study over a range of infant and maternal characteristics at trial entry or short-term neonatal outcomes (see web table S1).

In those who were followed up, the two study groups were balanced over a range of social and demographic measures (see web tables S2 and S3). The index of multiple deprivation score^14^ for each family showed a median value of 19 (IQR: 10, 31) for families in the GM-CSF arm compared with 19 (IQR: 10, 34) in the control arm.

Primary outcome: Kaufmann MPC scores for the GM-CSF group were 94 (SD 16) compared with 95 (SD 15) in controls, difference in means: −1 (95%CI −6 to 4). Subscale scores did not vary significantly between groups and the proportion scoring −2 SD or lower (<70) or between −1 and −2SD were similar (table 1). There were no significant differences across NEPSY scaled scores (table 1), and no significant differences in behavioural scores (table 2), nor in parent reported language and communication skills (table 3), between the two arms. In the GM-CSF and control groups, and despite mean IQ scores of 95, a large but similar proportion of each trial group were in receipt of special needs support (41% and 35%, respectively), statements of educational needs (17% vs 13%), and had met Key Stage 1 national attainment levels (table 4). Growth measures, expressed as Z or SD scores, were similar between the two groups (table 5).

**Table 1:** Measured cognitive outcomes for children exposed to GM-CSF compared with control children in the PROGRAMS trial groups at 5 years of age

	GM-CSF (n=79)		Control (n=78)		
	Mean or no/total	% or SD	Mean or no/total	% or SD	Difference in means (95% CI)
*Cognitive development*					
Kaufmann ABC					
Mental processing composite	94	(16)	95	(15)	−1 (−6 to 4)
<70	4/78	5%	2/77	3%	
**70–85**	9/78	12%	18/77	23%	
Simultaneous processing	95	(17)	95	(16)	0 (−5 to 5)
<70	6/78	8%	2	3%	
**70–85**	10/78	13%	20	26%	
Sequential processing	94	(19)	98	(14)	−3 (−8 to 2)
<70	7/78	9%	2	3%	
**70–85**	4/78	5%	13	17%	
Maths score	99	(14)	97	(13)	1 (−3 to 5)
<70	3/78	4%	5	6%	
**70–85**	8/78	10%	10	13%	
Riddles	94	(14)	93	(13)	1 (−3 to 5)
<70	5/76	7%	2/77	3%	
**70–85**	11/76	14%	18/77	23%	

Neuropsychological assessment (NEPSY)	Median	(IQR)	Median	(IQR)	Difference in medians (95% CI)

Sensorimotor component scaled scores					
Imitating hand positions	7	5, 8	7	5, 9	−0.2 (−1.1 to 0.8)
Visuomotor precision	6	4, 9	7	5, 8	−0.4 (−1.5 to 0.6)
Visuospatial component scaled score (2 of 4)					
Block construction	8	7, 10	8	5, 10	0.9 (−1.6 to 3.5)
Design copying	8	6, 10	8	6, 10	0.1 (−0.9 to 1.1)
Attention executive component (2 of 6)					
Visual attention *scaled score*	10	8, 12	10	8, 12	0.1 (−3.3 to 3.5)
Statue *total score*	23	14, 27	23	12, 27	−0.2 (−4.1 to 4.1)
Language component scaled score (2 of 7)					
Phonological processing	8	7, 10	9	6, 10	0.3 (−0.9 to 1.4)
Comprehension of instructions	10	8, 12	10	8, 11	−0.9 (−3.5 to 1.6)
Memory and learning component scaled score (4 of 5)					
Delayed memory for faces	9	5, 13	10	4, 12	0.1 (−1.2 to 1.9)
Narrative memory	8	6, 10	8	6, 10	0.3 (−0.8 to 1.4)
Delayed memory for names	7	2, 9	8	3, 10	−1.1 (−2.5 to 0.23)
Sentence repetition	9	7, 11	9	7, 11	0.9 (−1.6 to 3.4)

GM-CSF, granulocyte macrophage colony stimulating factor.

**Table 2:** Behaviour scores from the Strengths and Difficulties Questionnaire for children exposed to GM-CSF compared with control children in the PROGRAMS trial groups at 5 years

	GM-CSF	Control	Difference in medians (95% CI)
	Median (IQR)		
Parent SDQ	(n=62)	(n=54)	
Emotional disorder score (max. 15)	6 (6, 7)	6 (5, 7)	0 (0 to 1)
Disorder n (%)	0 (0%)	0 (0%)	
Conduct disorder score (max 15)	5 (5, 5)	5 (5, 6)	0 (0 to 0)
Disorder n (%)	0 (0%)	1 (2%)	
Hyperactivity disorder score (max 15)	5 (4, 6)	5 (4, 6)	0 (0 to 1)
Disorder n (%)	40 (65%)	38 (70%)	
Peer relationships score (max 15)	5 (5, 7)	5 (5, 7)	0 (0 to 0)
Disorder n (%)	1 (2%)	2 (4%)	
Overall score (max 60)	23 (20, 25)	21 (19, 24)	1.0 (−0.3 to 2.2)
Disorder n (%)	0 (0%)	0 (0%)	
Teacher SDQ	(n=73)	(n=70)	
Emotional disorder score (max. 15)	6 (5, 7)	6 (5, 8)	0 (0 to 0)
Disorder n (%)	0 (0%)	0 (0%)	
Conduct disorder score (max 15)	5 (4, 5)	5 (5, 5)	0 (0 to 0)
Disorder n (%)	0 (0%)	0 (0%)	
Hyperactivity disorder score (max. 15)	5 (4, 6)	5 (4, 6)	0 (-1 to 0)
Disorder n (%)	40 (55%)	34 (49%)	
Peer relationships score (max 15)	5 (4, 5)	5 (4, 5)	0 (0 to 0)
Disorder n (%)	10 (14%)	2 (3%)	
Overall score (max 60)	21 (19 to 23)	21 (20 to 23)	−0.5 (−1.4 to 0.5)
Disorder n (%)	0 (0%)	0 (0%)	
School adaptation (impact)	3 (2, 4)	3 (2, 4)	0 (0 to 1)

‘Disorder’ indicates the number of children with borderline or clinical scores compared with reference data.

GM-CSF, granulocyte macrophage colony stimulating factor; SDQ, Strengths and Difficulties Questionnaire.

**Table 3:** Scales derived from the Twins and Early Development Study (TEDS) measures at 4 years applied to the children exposed to GM-CSF compared with control children in PROGRAMS trial groups at 5 years of age

		GM-CSF (n=62)		Control (n=54)	
Outcome	Max score	No or median	% or IQR	No or median	% or IQR
Your child's development					
Talking (1–6), median (IQR)		6	6, 6	6	6, 6
Actions or words—n% with actions		3/61	5%	3	6%
Sounds younger—n%		17	27%	8	15%
Your child's words (total n)	48	41	29, 46	41	33, 48
Using language (total ‘yes’ responses)	14	12	7, 13	13	11, 14
Books (total ‘yes’ responses)	12	8	6, 9	8	6, 9
Communication worries n (%)		23	37%	14	26%
Professional advice n (%)		32	52%	24	44%
Communication score, median (IQR)	30	26	22, 26	26	24, 27
Total ‘TEDS’ score, median (IQR)	94	85	66, 93	86	80, 93

GM-CSF, granulocyte macrophage colony stimulating factor.

**Table 4:** School attainment for children exposed to GM-CSF compared with control children in PROGRAMS trial groups at 5 years of age

	GM-CSF (n=73)	Control (n=70)	Risk ratio (95% CI)
Special educational needs			
Academic	29/69 (42%)	24/65 (37%)	1.1 (0.7 to 1.7)
Behavioural	16/71 (23%)	11/61 (18%)	1.3 (0.6 to 2.5)
Statement (yes/no)*	12/72 (17%)	9/68 (13%)	1.3 (0.6 to 2.8)
On register (England only)†	26/67 (39%)	22/66 (33%)	1.2 (0.7 to 1.8)
Stage 1	1	2	
Stage 2	2	1	
Stage 3	1	2	
Stage 4/5	6	3	
Special needs support (yes/no)	31 (41%)	24/69 (35%)	1.2 (0.8 to 1.9)
Individual special needs plan	23	21	
One-to-one provision	15	6	
Small group provision	20	18	
Outreach teachers	5	3	
Educational psychologist	8	4	
Clinical psychologist	1	1	
Physiotherapist	2	5	
Speech/language therapist	12	14	
Requires additional support (yes/no)‡	19/71 (27%)	21 (30%)	0.9 (0.5 to 1.5)
Individual special needs plan	7	10	
One-to-one provision	8	10	
Small group provision	6	10	
Outreach teachers	1	2	
Educational psychologist	0	5	
Clinical psychologist	0	1	
Physiotherapist	2	1	
Speech/language therapist	4	9	

Key stage attainment	Median grade (IQR)		Difference in medians (95% CI)

English			
Speaking and listening	3 (3, 4)	3 (3, 4)	0 (0 to 1)
Reading	3 (3, 4)	3 (3, 4)	0 (0 to 0)
Writing	4 (3, 5)	3 (3, 4)	0 (0 to 1)
Spelling	3 (3, 5)	3 (3, 4)	0 (0 to 1)
Maths			
Using and applying	3 (3, 4)	3 (3, 4)	0 (0 to 0)
Numbers and algebra	3 (3, 4)	3 (3, 4)	0 (0 to 1)
Shape, space and measures	3 (3, 4)	3 (3, 4)	0 (0 to 0)

*Formal binding statutory educational statement of needs.

†Indicates progress through the statement process.

‡Teacher assessed need for support not provided.

GM-CSF, granulocyte macrophage colony stimulating factor.

**Table 5:** Growth at 5 years for children exposed to GM-CSF compared with control children in PROGRAMS trial groups at 5 years of age

	Mean (SD)		
	GM-CSF (n=60)	Control (n=61)	Difference in medians (95% CI)
Height			
cm	104 (10)	105 (9)	−0.9 (−4.4 to 2.7)
SDS*	−1.7 (2.4)	−1.6 (2.1)	−0.1 (−0.9 to 0.7)
Weight			
kg	17 (3)	17 (3)	0.3 (−0.7 to 1.3)
SDS*	−1.1 (1.3)	−1.3 (1.3)	0.2 (−0.3 to 0.6)
Head circumference			
cm	50 (2)	50 (2)	0 (−1 to 1)
SDS*	−1.7 (1.4)	−1.9 (1.3)	0.2 (−0.3 to 0.7)

*Height, weight, head circumference SD scores from UK-WHO standards.

GM-CSF, granulocyte macrophage colony stimulating factor.

Fewer families returned health questionnaires than the number of children who were assessed at school: 62 in the GM-CSF group and 54 in the control group (see web table S4). Admissions to hospital were infrequent in both groups and no child was admitted to an intensive care unit and mechanically ventilated. At 5 years the point prevalence of cough and wheeze were higher and the use of respiratory medications more frequent in the GM-CSF group (with relative risk values of 1.7–2 for most comparisons, web table S5), but none of the comparisons would be statistically significant if adjusted for multiple comparisons.

## Discussion

This is the first report of childhood outcomes at 5 years of age following the neonatal administration of GM-CSF to SGA very preterm babies in a randomised trial. We have previously reported that GM-CSF raises neonatal neutrophil counts, but does not appear to reduce the prevalence of neonatal sepsis or other neonatal morbidities; nor affect neurodevelopment at 2 years of age adjusted for prematurity.^6^ Here we show that it does not affect neuropsychological outcomes at 5 years. In contrast to the neonatal findings of marginally worse respiratory outcomes in the control babies, follow-up at 2 years and 5 years seems to indicate marginally worse respiratory outcomes among the GM-CSF treated infants though these differences were not statistically significant.

For the 5-year outcomes we achieved reasonable follow-up rates of 76%. Surviving children who were not evaluated did not differ over a range of perinatal and neonatal variables from children evaluated as part of this study. Nonetheless children who are not available for follow-up may have higher rates of impairment compared with those evaluated,^15^ ^16^ which may bias the results. We chose not to correct for preterm birth in calculating the age at assessment, as this is conventional at this age. The gestational age distributions were well balanced between the two groups and it would have been unlikely to alter the findings.

We chose a wide range of neuropsychological tests from direct observation, parents and teachers to make a comprehensive assessment. Furthermore we obtained summary functional information concerning educational attainment to confirm the results of our testing. Although the general cognitive scores appear only mildly depressed compared with standardisation data (5–6 points or approximately 0.33 SD), the test used was standardised in the late 1970s.^7^ The results of population testing of IQ tend to rise over time;^17^ using the original test the classmate comparison group used in the EPICure study at 6 years in 2001–2002 produced mean scores of 107 (SD 12) in term-born children.^18^ Hence the PROGRAMS group scores represent approximately −1 SD (13–14 points) below the mean at best, which is consistent with population estimates of scores in very preterm children. This is also in keeping with the teachers’ reports that 39% of the PROGRAMS group was receiving special educational support and 15% had an educational statement of needs in the 1st year of schooling.

In the absence of a comparison group of babies born at full term it is difficult to evaluate the performance of this group of SGA children relative to other very preterm groups. The majority of children in this study had fetal growth restriction. In a prospective cohort study of children identified before birth with growth restriction, scores at 6 years were on average 11 points lower compared with AGA-matched children, whose IQ scores were 12 points lower than term-born children.^19^ At 2 years of age adjusted for prematurity Bayley MDI (BSID-II) scores for the PROGRAMS cohort were lower than expected considering their gestational age at birth and scores were similar to those from our national cohort of babies born before 26 weeks of gestation (EPICure).^20^ At the 5-year assessment the scores using the same test appear higher, suggesting some catch up by the PROGRAMS group. However, in comparison to national data from Scotland, where the proportion with special educational needs among very preterm survivors rises from 12% for those born at 30 weeks gestation to around 30% for those born at 26 weeks gestation,^21^ the rate of 39% reported in this study seems high. It seems likely that SGA very preterm children have higher rates of cognitive and learning problems than their appropriately grown peers. Thus, it is important to identify very preterm children to teachers for close assessment of their needs.

The persistence of the trend for increased risk of respiratory symptoms is intriguing. Cohort studies associate bronchopulmonary dysplasia with perinatal infection^22^ and fetal growth restriction.^23^ Further the associations between measured inflammatory mediators and bronchopulmonary dysplasia or developmental outcomes seem particularly strong when these mediators are elevated postnatally,^24^ over the same period as when GM-CSF was administered in this study. Nonetheless we found these findings difficult to explain at 2 years. We did not collect information on family smoking status, which may have accounted for this difference. The inconsistency between outcomes at discharge, and at 2 years and 5 years suggests these findings may have arisen by chance.

Prior to the clinical use of G-CSF and GM-CSF in newborn babies during the 1990s, concern was raised that exposure of the immature haematopoietic and immune system to pharmacological doses of these recombinant human cytokines might lead to later alternations to haematopoiesis, immune function, or drive the emergence of malignant clones and leukaemia.^25^ Follow-up of an early G-CSF study in neonates found no alteration in routine haematological or biochemical parameters at 2 years of age;^26^ there have been no cases of leukaemia in the PROGRAMS cohort, and the equivalent number of non-surgical admission to hospital between groups (see web table S4). These observations provide no evidence to suggest altered defence against bacterial or viral infection. Safety is further supported by the now extensive experience of long-term use of CSF therapy in children with cyclical neutropenia.^27^

## Conclusions

When evaluated in a randomised trial, with long-term follow-up at 2 years and 5 years of age, GM-CSF does not appear to offer significant benefit in terms of neuroprotection.

## Supplementary Material

Web supplement
