# Supplementary material for: A randomised trial of granulocyte-macrophage colony-stimulating factor for neonatal sepsis: childhood outcomes at 5 years
Source: Arch Dis Child Fetal Neonatal Ed. 2015 Apr 28;100(4):F320–6. doi: 10.1136/archdischild-2014-307410 (PMC4484494; doi:10.1136/archdischild-2014-307410)
Supplement: Web supplement [file fetalneonatal-2014-307410-s1.pdf]

Web appendix for:

A randomised trial of granulocyte-macrophage colony-stimulating factor for neonatal sepsis: Childhood outcomes at 5 years

Neil Marlow, Timothy Morris, Peter Brocklehurst, Robert Carr, Frances Cowan, Nishma Patel, Stavros Petrou, Margaret Redshaw, Neena Modi, Caroline J Doré

Correspondence to:

Neil Marlow
Professor of Neonatal Medicine
UCL Institute for Women's Health
74 Huntley Street
London WC1E 6AU
United Kingdom

Tel: +44 20 7679 0834
Fax: +44 20 3108 2036
Email: n.marlow@ucl.ac.uk

Web table S1: Comparison of infant and maternal characteristics at trial entry and short term outcomes between surviving children who were or were not followed up for PROGRAMS trial groups at 5 years of age

	**Followed up (*n* = 164)**		**Not followed up (*n* = 52)**		Difference or relative risk (RR)	95% CI for difference or RR
	n or median	% or IQR	*n* or median	% or IQR		
**Characteristics at trial entry**						
Male, n (%)	79	48%	23	44%	1.1	0.7 to 1.5
Age at recruitment, h, median (IQR)	45	26, 63	41	22, 55	4.4	-1.7 to 11.0
Gestational age, weeks, median (IQR)	29	28, 30	29	27, 30	0	-0.6 to 0.4
Birthweight, g, median (IQR)	805	646, 933	840	718, 951	-37	-95 to 20
Neutrophil count at trial entry, $x10^9/L$, median (IQR)	2.8	1.3, 4.3	2.0	1.2, 3.7	0.3	-0.3 to 0.9
Neutropenia (<$1.1x10^9/L$) at trial entry, n (%)	26	16%	9	17%	0.9	0.5 to 1.8
Multiple pregnancies, n (%)	40	24%	13	25%	1.0	0.6 to 1.7
Maternal antenatal steroids administered 4 h or more before delivery, n (%)	129/139	93%	45/47	96%	1.0	0.9 to 1.1
Infants given surfactant within 4 h of birth, n (%)	115/126	91%	37/42	88%	1.0	0.9 to 1.2
Cranial ultrasound findings at trial entry						
Normal	128/158	81%	42	81%		
Minor abnormality	26/158	16%	7	13%		
Severe abnormality	4/158	3%	3	6%		
**Short term outcomes**						
Necrotising enterocolitis (confirmed at surgery or post mortem)	5	3%	1	2%	1.6	0.2 to 13.3
Sepsis: culture positive to day 28 from trial entry	41	25%	10	19%	1.3	0.7 to 2.4
Sepsis: culture positive and probable to day 28	51	31%	17	33%	0.9	0.6 to 1.5
Cranial ultrasound findings						
Normal	108/138	78%	31/45	69%		
Minor abnormality	20/138	15%	10/45	22%		
Severe abnormality	10/138	7%	4/45	9%		
Oxygen dependency at day 28, n (%)	102	62%	29	56%	1.1	0.9 to 1.5
Oxygen dependency at 36 weeks PMA, n (%)	79	48%	21	40%	1.2	0.8 to 1.7

For categorical variables summary measures are number, %, relative risk and 95% confidence interval (CI).
For continuous variables summary measures are median, interquartile range (IQR), difference in medians and 95% CI.
The denominator is the entire population, unless otherwise stated.

Web table S2: Infant and maternal characteristics at trial entry and short term outcomes for all children followed up for PROGRAMS trial groups at 5 years of age.

	GM-CSF (*n* = 83)		Control (*n* = 81)			
	n or median	% or IQR	*n* or median	% or IQR	Difference or relative risk (RR)	95% CI for difference or RR
**Characteristics at trial entry**						
Male, *n* (%)	39	47%	40	49%	1.0	(0.7 to 1.3)
Age at recruitment, h, median (IQR)	46	24, 53	46	27, 64	1	(-3 to 9)
Gestational age, weeks, median (IQR)	29	27, 30	29	28, 30	-0.1	(-0.7 to 0.3)
Birthweight, g, median (IQR)	830	655, 930	785	625, 955	-10	(-69 to 48)
Neutrophil count at trial entry, $x10^9$/L ,median (IQR)	2.7	1.3, 4.4	2.8	1.3, 4.3	0.1	(-0.5 to 0.8)
Neutropenia (<$1.1x10^9$/L) at trial entry, *n* (%)	14/80	18%	12/76	16%	1.1	(0.5 to 2.2)
Multiple pregnancies, *n* (%)	23	27%	17	21%	1.3	(0.8 to 2.3)
Maternal antenatal steroids administered 4h or more before delivery, *n* (%)	63/70	90%	66/69	96%	0.94	(0.9 to 1.0)
Infants given surfactant within 4h of birth, *n* (%)	58/65	89%	57/61	93%	1.0	(0.9 to 1.1)
Cranial ultrasound findings at trial entry						
Normal	64/82	78%	64/76	84%		
Minor abnormality	17/82	21%	9/76	12%		
Severe abnormality	1/82	1%	3/76	4%		
**Short term outcomes**						
Necrotising enterocolitis (confirmed at surgery or post mortem)	2	2%	3	4%	0.7	(0.1 to 3.8)
Sepsis: culture positive to day 28 from trial entry	24	29%	17	21%	1.4	(0.8 to 2.4)
Sepsis: culture positive and probable to day 28	25	30%	26	32%	0.9	(0.6 to 1.5)
Cranial ultrasound findings						
Normal	57/66	86%	51/72	71%		
Minor abnormality	5/66	8%	15/72	21%		
Severe abnormality	4/66	6%	6/72	8%		
Oxygen dependency at day 28, *n* (%)	57	69%	45	56%	1.2	(1.0 to 1.6)
Oxygen dependency at 36 weeks PMA, *n* (%)	45	54%	34	42%	1.3	(0.9 to 1.8)

For categorical variables summary measures are number, %, relative risk and 95% confidence interval (CI).
For continuous variables summary measures are median, interquartile range (IQR), difference in medians and 95% CI.
The denominator is the entire population, unless otherwise stated.

Web table S3: Parental information (taken from 2y assessment) based on population examined in PROGRAMS trial groups at 5 years of age

Variable	GM-CSF (*n* = 62) No.		Control (*n* = 66) No.	
Marital status				
Married	44	71%	40	60%
Living with partner	11	18%	17	26%
Single	7	11%	14	14%
Biological parents at home	52/61	85%	55	83%
Housing				
Home owner	40/51	78%	38/52	73%
Rooms in home median (IQR)	5	4, 6	5	3,6
Use of telephone	60	97%	63	95%
Use of Car	56	90%	55	83%
Mother's educational level				
O'Level/GCSE/equivalent or lesser*	30	48%	26	39%
Parental employment status				
Mother working	31/61	51%	32/64	50%
Mother in full time education	0	0%	2	3%
Mother not working or in full time education	30	48%	32	48%
Father working	51/57	89%	54/60	90%
Social index (median (IQR))	***n* = 52**		***n* = 47**	
Index of multiple deprivation (2007)	19	10, 31	19	10, 34
Income deprivation affecting children index	0.21	0.09, 0.41	0.29	0.12, 0.45

*Notes: * qualifications for school leaver at age 16 in English school (GCSE: General Certificate of Secondary Eduaction); note this excludes higher educational progression*

Web table S4: Hospital admissions over the past 12 months for PROGRAMS trial groups at 5 years of age

**Outcome**	**GM-CSF (*n* = 62)**	**Control (*n* = 54)**	Risk ratio (95% CI)
Respiratory admission (*n*)	3 (5%)	5 (9%)	0.5 (0.1 to 2.1)
Median* (Upper Quartile; Max)	1 (1; 1)	1 (1; 1)	
Surgical admission (*n*)	9 (14%)	5 (9%)	1.6 (0.6 to 4.4)
Median* (Upper Quartile; Max)	1 (2; 2)	1 (1; 1)	
ICU admission and mechanically ventilated (*n*)	0 (0%)	0 (0%)	–
Median* (Upper Quartile; Max)	–	–	–
Other admission (*n*)	3 (5%)	4 (7%)	0.7 (0.2 to 2.8)
Median* (Upper Quartile; Max)	1 (2; 2)	2 (4; 4)	

Notes: *number of admissions among those who had had an admission

Web table S5: Respiratory Outcomes for PROGRAMS trial groups at 5y

Outcome		GM-CSF (*n* = 62) No.	GM-CSF (*n* = 62) %	Control (*n* = 54) No.	Control (*n* = 54) %	RR 95% CI for worst adverse outcome
**Chest symptoms**						
Cough						
> once a week		9	14%	12	22%	
Weekly-monthly		13	21%	4	7%	
Once a month or less		7	11%	6	11%	
Cough with infection only		16	26%	7	13%	2.0 (0.9 to 4.5)
Cough on exercise		7	11%	3	6%	2.0 (0.6 to 7.5)
Disturbed sleep		29	47%	29	37%	1.3 (0.8 to 2.0)
Dry night time cough		28	45%	17	31%	1.4 (0.9 to 2.3)
Wheeze						
> once a week		1	2%	2	4%	
Weekly-monthly		6	10%	2	4%	
Once a month or less		10	16%	2	4%	
Wheeze with infection only		8	13%	4	7%	1.7 (0.6 to 5.5)
Wheeze on exercise		4	6%	2	4%	1.7 (0.3 to 9.1)
Disturbed sleep		5	9%	12	19%	2.1 (0.8 to 5.6)
**Chest treatments**						
Antibiotics		36	58%	22	41%	1.4 (1.0 to 2.1)
Oxygen		2	3%	1	2%	1.7 (0.2 to 18.7)
Bronchodilators	Current	9	15%	4	7%	2.0 (0.6 to 6.0)
	Last 12mo	24	39%	12	22%	1.7 (1.0 to 3.1)
Inhaled steroids	Current	6	10%	3	6%	1.7 (0.5 to 6.6)
	Last 12mo	20	32%	6	11%	2.9 (1.3 to 6.7)
Oral steroids	Current	0	0%	1	2%	–
	Last 12mo	3	5%	3	6%	0.9 (0.2 to 4.1)
